# Cost-effectiveness of single-dose versus two-dose HPV vaccination: a Markov cohort modelling analysis of a Kenya–India LMIC composite

**DOI:** 10.3389/fpubh.2026.1833764

**Published:** 2026-06-04

**Authors:** Jinyao Wang, Xiaolan Wang, Jie Xu, Qiuxiang Xu

**Affiliations:** 1 Department of Clinical Medicine, YunCheng Vocational Nursing College, YunCheng, China; 2 Department of Basic Medicine, Luohe Medical College, Luohe, China; 3 Department of Anesthesiology and Surgery, Shanxi Provincial People’s Hospital, Taiyuan, China; 4 Department of Pediatric Emergency, Shanxi Provincial Children’s Hospital, Taiyuan, Shanxi, China

**Keywords:** cervical cancer, cost-effectiveness analysis, disability-adjusted life years, HPV vaccination, India, Kenya, low- and middle-income countries, Markov model

## Abstract

**Background:**

Cervical cancer imposes a disproportionate burden in low- and middle-income countries (LMICs), where second-dose dropout substantially limits the real-world effectiveness of two-dose HPV vaccination programmes. Single-dose schedules eliminate dropout but provide shorter-duration protection. Whether single-dose vaccination is economically preferred to two-dose vaccination, and under what conditions the two strategies yield divergent recommendations depending on the health outcome metric used, has received limited attention in published cost-effectiveness analyses, particularly for the direct head-to-head schedule comparison.

**Methods:**

We developed a static Markov cohort model, assessed for face validity against observed incidence data, tracking 100,000 girls aged 9 years through a lifetime horizon (to age 99) within an LMIC composite derived from Kenya (low-income) and India (lower-middle-income). Three strategies were compared: no vaccination (reference), 1-dose HPV vaccination (efficacy 87% against HPV 16/18-attributable CIN2+; 20-year base-case protection duration), and 2-dose vaccination (efficacy 93%; lifetime protection). Programme coverage was 49% for both strategies; a 15% second-dose dropout rate reduced 2-dose effective coverage to 41.7%. Costs (2024 USD), disability-adjusted life years (DALYs) averted, and incremental cost-effectiveness ratios (ICERs) were estimated from a healthcare payer perspective with 3% annual discounting, within a static direct-effect framework that does not capture herd immunity. Willingness-to-pay thresholds of USD 200/DALY (low-income) and USD 500/DALY (lower-middle-income) were applied from Disease Control Priorities, 3rd edition. Uncertainty was characterised through 10,000-simulation probabilistic sensitivity analysis (PSA) and pre-specified scenario analyses examining second-dose dropout rates from 5 to 45% and 1-dose protection durations of 10 years, 20 years (base-case), and lifetime.

**Results:**

Both strategies were dominant (cost-saving and more effective) versus no vaccination. Per 100,000 girls, 1-dose prevented 64 invasive cervical cancer (ICC) cases and 77 deaths, averting 5,545 DALYs with a net cost saving of USD 1,233,242 (ICER: −USD 222/DALY). Two-dose prevented more cases (87) and deaths (95) but, under the base-case 20-year protection assumption, averted fewer DALYs (5,149) and saved less (USD 980,690). This divergence reflects the age-concentration of 1-dose protection in women aged 15–29 years (86.4 DALYs per averted case vs. 59.2 for 2-dose) and is structurally dependent on the 20-year protection duration assumption; at lifetime protection, this DALY efficiency difference diminishes. In PSA, 1-dose was dominant in 99.3% of simulations (mean ICER: −USD 195/DALY; 95% credible interval [CrI]: −USD 315 to −USD 64) and cost-effective at both thresholds in 100% of simulations. Two-dose was dominant in 91.8% of simulations (mean ICER: −USD 133/DALY; 95% CrI: −USD 284 to +USD 136). The 1-dose ICER was invariant to second-dose dropout rate; the 2-dose ICER deteriorated from −USD 141/DALY at 5% dropout to −USD 105/DALY at 45% dropout.

**Conclusion:**

The finding that the single-dose strategy achieves greater DALY efficiency per dollar is structurally dependent on the base-case assumption of 20-year single-dose protection duration and should not be interpreted as a general empirical finding. Under this specific assumption, elimination of second-dose dropout raised effective coverage from 41.7 to 49.0% and concentrated protection in women aged 15–29 where each averted case contributes more years of life lost. Sensitivity analyses show this DALY efficiency advantage narrows or reverses if single-dose protection is shorter (10 years) or extends to lifetime. Across all scenarios examined, the two-dose strategy prevented more absolute cancer cases and deaths than single-dose. These are scenario-specific, exploratory findings generated within a Kenya–India composite under a static direct-effect framework; national programme decisions should incorporate country-specific dropout rates, emerging long-term evidence on single-dose protection duration, locally applicable outcome metrics, and country-calibrated modelling before drawing policy conclusions.

## 1 Introduction

Cervical cancer remains a leading cause of cancer mortality among women in low- and middle-income countries (LMICs). In 2022, an estimated 661,000 new cases and 349,000 deaths were attributed to cervical cancer globally, with the large majority of this burden concentrated in LMICs where organised screening programmes have limited reach (1). Age-standardised incidence rates in high-burden settings—Kenya (32.8 per 100,000) and India (17.7 per 100,000)—substantially exceed rates in high-income countries, reflecting persistent inequities in both primary prevention and early detection. The WHO global strategy to accelerate cervical cancer elimination (2) sets a 90–70-90 target framework: vaccinating 90% of girls by age 15, screening 70% of women by ages 35 and 45, and treating 90% of identified disease, placing HPV vaccination as the cornerstone of global cancer control.

Prophylactic HPV vaccines have demonstrated high efficacy against the HPV 16/18 genotypes responsible for approximately 70% of cervical cancers worldwide. The standard two-dose schedule, recommended for girls aged 9–14 years prior to sexual debut, has proven challenging to complete in many LMIC programmatic contexts. Health system capacity constraints, cold-chain requirements, the logistical burden of a second clinic visit, and competing health priorities contribute to substantial second-dose dropout. This attrition directly reduces effective population coverage and compromises programme cost-effectiveness: a 15% dropout rate—a conservative estimate for many settings—reduces effective two-dose coverage from a 49% target to 41.7%, a gap of more than 7 percentage points relative to a single-dose schedule requiring no return visit.

Evidence supporting single-dose HPV vaccination has expanded substantially in recent years. The KEN SHE randomised controlled trial, conducted among Kenyan girls and young women aged 15–20 years, demonstrated 97.5% vaccine efficacy against persistent HPV 16/18 infection at 18 months following a single dose of the nonavalent vaccine (3). Longer-term immunogenicity data from the Costa Rica HPV4 Trial suggest durable antibody responses following single-dose vaccination, supporting the biological plausibility of extended protection (4). In 2022, the WHO Strategic Advisory Group of Experts on Immunization (SAGE) issued a recommendation that a single-dose schedule is an acceptable alternative to two-dose schedules for girls aged 9–14 years, citing comparable efficacy with substantially improved programmatic feasibility (5). The duration of single-dose protection, however, remains incompletely characterised: current evidence extends to approximately 18 months, and longer-term estimates used in modelling studies represent extrapolations rather than established empirical findings.

The economic implications of this policy shift remain incompletely characterised for the direct comparison between single- and two-dose schedules. Published cost-effectiveness analyses have predominantly evaluated HPV vaccination against a no-vaccination comparator, with fewer studies quantifying the incremental economic value of choosing 1-dose over 2-dose. Among those that address the schedule comparison, the mechanism through which protection duration and effective coverage interact to determine DALY outcomes—and in particular, the conditions under which DALY-based and case-based metrics yield divergent strategy rankings—has received limited attention. This distinction has direct policy relevance: a decision-maker using absolute cancer cases as the primary metric may reach a different conclusion than one using DALYs averted, even when applied to the same model and the same data. Several recent modelling analyses have refined estimates of single-dose HPV vaccination impact in LMIC settings. Prem et al. (6) conducted a 188-country comparative modelling analysis of one-dose versus two-dose schedules, finding both strategies cost-saving across the majority of LMICs under opportunity-cost-based thresholds. Bénard et al. (7) ranked the most efficient HPV vaccination strategies across low-income and lower-middle-income countries, providing a framework for priority-setting. Mwenda et al. (8) modelled the impact, cost-effectiveness, and budget implications of Umutesi et al. (32) HPV vaccination specifically for Kenya, reporting dominant cost-effectiveness consistent with our findings. Termrungruanglert et al. (9) further compared single-dose and two-dose schedules of bivalent, quadrivalent, and nonavalent vaccines in an LMIC setting, finding single-dose schedules cost-effective across vaccine types. However, these analyses have predominantly evaluated vaccination against a no-vaccination counterfactual, and few have explicitly quantified how the DALY-based and case-based strategy comparisons between single- and two-dose schedules diverge as a function of protection duration assumptions.

We address these gaps by developing an exploratory, static Markov cohort model for a composite setting derived from Kenya and India—two countries representing distinct LMIC income categories (low-income and lower-middle-income, respectively) that have adopted or are actively scaling HPV vaccination programmes and have the country-specific epidemiological data required for model parameterisation. Because we use a static cohort model, our analysis captures only direct vaccine protection effects within a direct-effect framework; indirect effects through herd immunity are not modelled and our estimates represent conservative lower bounds on programme impact. We compare 1-dose vaccination, 2-dose vaccination, and no vaccination over a lifetime horizon, using opportunity-cost-based willingness-to-pay thresholds from Disease Control Priorities, 3rd edition. We characterise parameter uncertainty through 10,000-simulation probabilistic sensitivity analysis and systematic scenario analyses across second-dose dropout rates from 5 to 45% and 1-dose protection durations of 10 years, 20 years (base-case), and lifetime. We additionally provide an exploratory age-band decomposition of how the timing of cancer prevention—determined by protection duration—generates divergent DALY and case-count outcomes between the two schedules, offering a framework for metric-transparent policy communication.

## 2 Methods

### 2.1 Study design and setting

We conducted a static Markov cohort cost-effectiveness analysis (CEA) comparing three cervical cancer prevention strategies: no vaccination (reference), single-dose (1-dose) HPV vaccination, and two-dose (2-dose) HPV vaccination, from a healthcare payer perspective. Costs and health outcomes were discounted at 3% per annum. The study followed the Consolidated Health Economic Evaluation Reporting Standards (CHEERS) 2022 checklist (10). The primary setting was an LMIC composite derived as the simple average of epidemiological and health system parameters from Kenya and India, giving equal analytical weight to low-income and lower-middle-income country contexts. A completed CHEERS 2022 checklist (10), is provided in Supplementary File S1, with page and section references for each of the 28 reporting items. No item was omitted; items not applicable to static cohort modelling (e.g., characterisation of heterogeneity via subgroup analysis) are marked accordingly in the checklist.

### 2.2 Model structure

**Figure 1:**
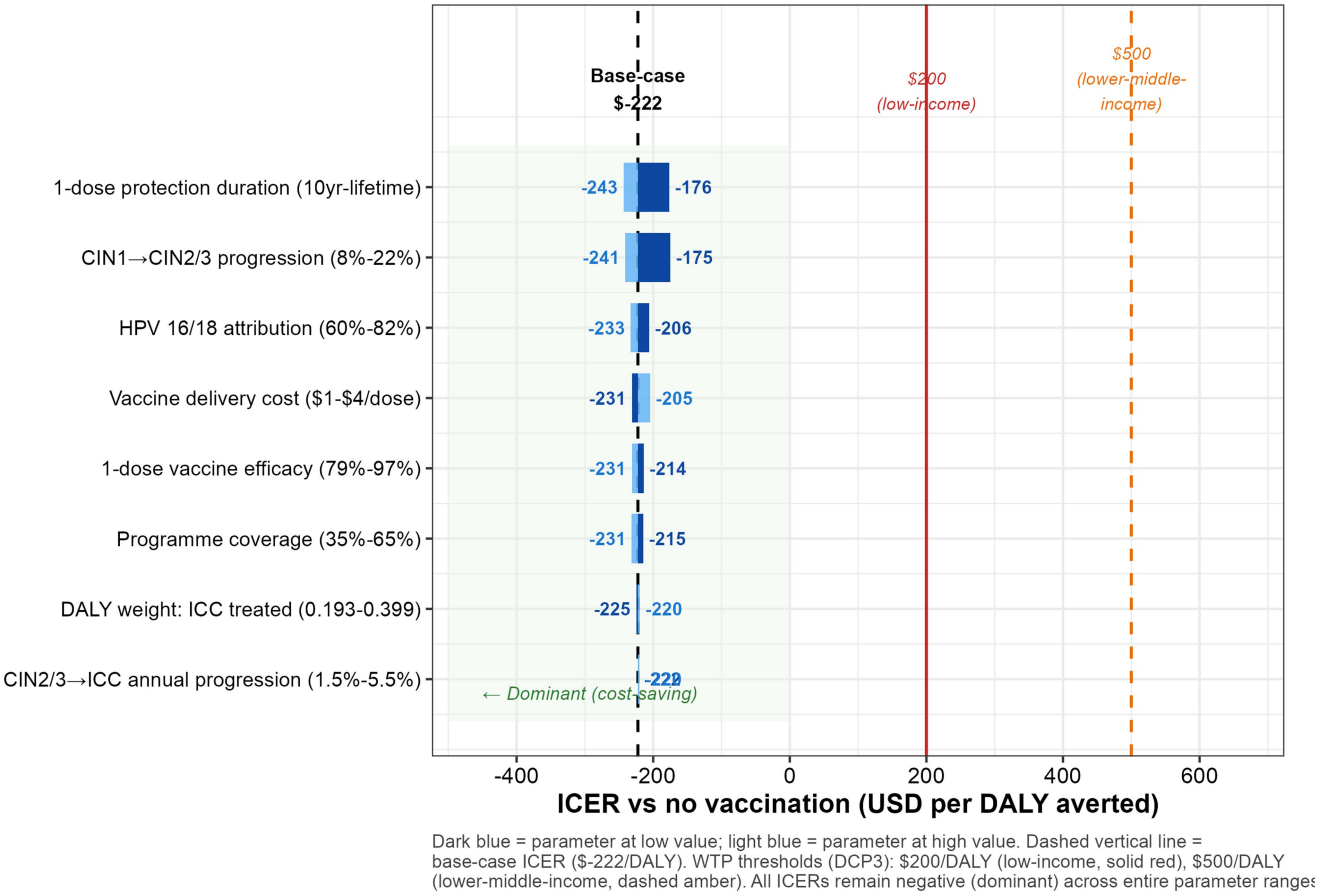
Markov model state-transition diagram. Health states (blue boxes), invasive cervical cancer stages (amber), recovered/cleared state (green), and absorbing death states (circles) are connected by annual transition probabilities listed in Table 1. Solid arrows denote disease-progression and regression transitions; dashed arrows denote age- and sex-specific background all-cause mortality from WHO Global Health Estimates 2021 life tables. The vaccination branch (dashed cluster) shows the two intervention strategies with their respective effective coverage (49.0% for 1-dose; 41.7% for 2-dose after 15% second-dose dropout) and vaccine efficacy against HPV 16/18–attributable CIN2+. CIN = cervical intraepithelial neoplasia; ICC = invasive cervical cancer; VE = vaccine efficacy.

The model tracked a hypothetical birth cohort of 100,000 girls aged 9 years through their lifetime (to age 99) using annual Markov cycles. Eleven mutually exclusive health states were included: susceptible, HPV-infected (high-risk types), CIN1, CIN2/3, invasive cervical cancer (ICC) by stage at diagnosis (Stages I–IV), cancer death, other-cause death, and recovered/cleared (Figure 1). Background all-cause mortality derived from WHO Global Health Estimates 2021 life tables was applied to all living states, allowing explicit modelling of competing risks (11). When the sum of competing transition probabilities within a health state exceeded 1.0 in any cycle, all probabilities were scaled proportionally to sum to 1.0. A static Markov cohort model was selected rather than a dynamic transmission model for three reasons: (i) the analytic focus is on direct comparison of vaccination schedules in terms of operational coverage and protection duration, rather than population-level transmission dynamics; (ii) country-specific sexual mixing, HPV type-specific force-of-infection, and screening cascade parameters required for dynamic model calibration are not available at comparable quality for both Kenya and India; and (iii) static models produce conservative, lower-bound estimates of programme impact because indirect protection via herd immunity is excluded. Dynamic HPV transmission models consistently yield larger estimates of prevented cases and DALYs averted at a given coverage level, typically 20–50% above the static direct-effect estimate at 40–50% coverage (12, 13). Our estimates should therefore be interpreted as a lower bound on programme impact, and the relative cost-effectiveness ranking of 1-dose vs. 2-dose reported here may differ under dynamic modelling if second-dose dropout increases transmission-cascade contribution from unvaccinated age cohorts.

### 2.3 Model calibration and validation

Under the no-vaccination scenario, the model projected 2,252 lifetime ICC cases per 100,000-girl cohort. Model face validity was assessed by comparing this projected lifetime incidence against an annualised rate derived from the GLOBOCAN 2022 age-standardised incidence figure for the LMIC composite, using a single-point consistency check. This does not constitute systematic internal or external validation; age-specific predicted incidence was not compared against observed age-specific data, and no structural validation against independent datasets was performed. Formal Bayesian calibration was not performed; natural history parameters were set to published point estimates with ranges explored in sensitivity analyses. Given the absence of formal calibration, the model outputs should be interpreted as indicative estimates derived from an exploratory policy analysis framework, rather than as predictions generated by a validated epidemiological model. Conclusions drawn from this model are conditional on the parameter assumptions described in Table 1 and should be re-evaluated as country-specific calibration data become available. In addition to the single-point consistency check against GLOBOCAN 2022 incidence, model face validity was further assessed by benchmarking base-case outputs against published HPV cost-effectiveness models applied to comparable LMIC settings. Specifically, per-100,000-cohort estimates of lifetime ICC cases under no vaccination and DALYs averted under 2-dose vaccination were compared against the 188-country modelling analysis by Prem et al. (6) and the PRIME model estimates for Kenya and India reported by Jit et al. (14) and by WHO-affiliated modelling analyses informing the WHO Strategic Advisory Group of Experts on Immunization 2022 single-dose recommendation (15). Our projected lifetime ICC incidence (2,252 per 100,000) falls within the range reported for East African and South Asian LMIC settings in these analyses (range: 1,900–2,600 per 100,000 under comparable vaccination-naive scenarios). A detailed comparison of model outputs against these published analyses is presented in Supplementary Table S4. This cross-model consistency check supports the face validity of the no-vaccination reference arm but does not constitute formal external validation; age-specific incidence was not benchmarked and natural history parameter uncertainty remains a limitation (Section 4.5).

**Table 1:** Model input parameters.

Parameter	Base-case	Range	PSA distribution	Source
Epidemiological parameters				
Cervical cancer incidence ASR (per 100,000 women)—Kenya	32.8	28–38	Gamma	GLOBOCAN 2022; Bray et al. (1)
Cervical cancer incidence ASR—India	17.7	14–22	Gamma	GLOBOCAN 2022
Cervical cancer incidence ASR—LMIC composite	25.3	20–31	Gamma	GLOBOCAN 2022
HPV 16/18 attribution fraction	0.72	0.60–0.82	Beta (*α* = 72, *β* = 28)	de Martel et al. (16)
Stage I at diagnosis	0.18	0.12–0.25	Dirichlet	Sankaranarayanan et al. (18)
Stage II at diagnosis	0.28	0.20–0.36	Dirichlet	Same
Stage III at diagnosis	0.37	0.28–0.46	Dirichlet	Same
Stage IV at diagnosis	0.17	0.10–0.24	Dirichlet	Same
Natural history transition probabilities (annual)				
HPV acquisition rate (peak, ages 15–24)	0.18	0.12–0.25	Beta	Clifford et al. (19)
HPV → CIN1 progression	0.30	0.20–0.40	Beta (*α* = 30, *β* = 70)	Myers et al. (22)
CIN1 → CIN2/3 progression	0.15	0.08–0.22	Beta (*α* = 15, *β* = 85)	Ostör (23)
CIN2/3 → ICC progression	0.035	0.015–0.055	Beta (*α* = 3.5, *β* = 96.5)	McCredie et al. (21)
HPV clearance (regression)	0.55	0.45–0.65	Beta (*α* = 55, *β* = 45)	Ho et al. (20)
CIN1 regression	0.40	0.30–0.50	Beta (*α* = 40, *β* = 60)	Ostör (23)
CIN2/3 regression	0.15	0.08–0.25	Beta (*α* = 15, *β* = 85)	Ostör (23)
Vaccine parameters				
2-dose vaccine efficacy vs. HPV 16/18 (CIN2 + endpoint)	0.93	0.88–0.97	Beta (*α* = 93, *β* = 7)	Lehtinen et al. (29); (30)
1-dose vaccine efficacy vs. HPV 16/18	0.87	0.79–0.97	Beta (*α* = 87, *β* = 13)	Barnabas et al. (3)
Programme coverage (2-dose target)	0.49	0.35–0.65	Beta	WHO-UNICEF WUENIC (33)
Second-dose dropout rate	0.15	0.05–0.45	Beta	Ladner et al. (17)
2-dose effective coverage [coverage × (1 − dropout)]	0.417	—	Derived	—
1-dose effective coverage (no dropout)	0.490	—	Derived	—
2-dose duration of protection	Lifetime	—	Deterministic	WHO (5)
1-dose duration of protection (base-case)	20 years	10 yr–lifetime	Deterministic (SA only)	Barnabas et al. (3); Kreimer et al. (4)
5-year survival by stage at diagnosis (LMIC)				
Stage I	0.75	0.65–0.85	Beta	Sankaranarayanan et al. (18)
Stage II	0.50	0.40–0.62	Beta	Same
Stage III	0.25	0.15–0.38	Beta	Same
Stage IV	0.08	0.03–0.15	Beta	Same
Costs (2024 USD)				
Vaccine unit price (per dose)	$4.50	$2.25–$9.00	Deterministic (SA only)	UNICEF (24)
Vaccine delivery cost (per dose)	$2.00	$1.00–$4.00	Gamma (shape = 4, rate = 2)	Slavkovsky et al. (25)
CIN2/3 treatment cost	$75	$40–$120	Gamma	Lince-Deroche et al. (26)
ICC treatment cost—Stage I	$1,050	$700–$1,600	Gamma	Insinga et al. (35) (LMIC-adapted)
ICC treatment cost—Stage II	$2,450	$1,500–$3,500	Gamma	Same
ICC treatment cost—Stage III	$3,050	$2,000–$4,500	Gamma (shape = 4, rate = 0.0013)	Same
ICC treatment cost—Stage IV (palliative)	$1,600	$900–$2,500	Gamma	Same
DALY weights and economic parameters				
Disability weight—CIN2/3	0.011	0.005–0.022	Beta (*α* = 1.1, *β* = 99.9)	GBD 2019 [Vos et al. (27)]
Disability weight—ICC treated	0.288	0.193–0.399	Beta (*α* = 28.8, *β* = 71.2)	GBD 2019
Disability weight—ICC terminal/palliative	0.451	0.307–0.600	Beta	GBD 2019
Life expectancy at birth—LMIC composite	68.0 yr	—	Deterministic	WHO Life Tables 2023
Discount rate	3%	0–5% (SA only)	Deterministic	WHO guidelines; Drummond et al. (34)
WTP threshold—low-income countries	$200/DALY	$100–$400	Deterministic (SA only)	Jamison et al. Jamison et al. (28)
WTP threshold—lower-middle-income countries	$500/DALY	$200–$800	Deterministic (SA only)	Jamison et al. (28)

ASR, age-standardised rate; CIN, cervical intraepithelial neoplasia; ICC, invasive cervical cancer; DALY, disability-adjusted life year; PSA, probabilistic sensitivity analysis; SA, scenario analysis; WTP, willingness-to-pay; GBD, Global Burden of Disease; DCP3, Disease Control Priorities 3rd edition. All costs expressed in 2024 USD. Beta distributions parameterised by alpha (mean numerator) and beta (mean denominator). Discount rate fixed at 3% per annum and not included in PSA. LMIC composite = simple average of Kenya and India parameters, giving equal analytical weight to low-income (Kenya) and lower-middle-income (India) country contexts.

### 2.4 Vaccination strategies

Vaccination was modelled as occurring at age 9 years, prior to sexual debut and HPV exposure (which peaks between ages 15 and 24). For the 1-dose strategy, base-case protection duration was 20 years from vaccination (expiring at age 29), selected as a conservative modelling choice in the absence of long-term efficacy data; scenario analyses explored 10-year and lifetime protection durations. For 2-dose, protection was assumed lifelong, consistent with the WHO 2022 HPV vaccine position paper.

Vaccine efficacy (VE) against HPV 16/18-attributable CIN2 + was 87% for 1-dose and 93% for 2-dose. The 1-dose VE was conservatively adjusted from the 97.5% (95% CI: 81.6–99.7%) infection-endpoint estimate in the KEN SHE trial (3) to reflect expected attenuation at CIN2 + endpoints. VE was applied to the 72% of ICC attributable to HPV 16/18 (16). Programme coverage was 49% for both strategies. A 15% second-dose dropout rate (17) reduced 2-dose effective coverage to 41.7% [= 0.49 × (1–0.15)]; 1-dose maintained 49.0% effective coverage. This 7.3 percentage-point difference in effective coverage is the primary structural driver of the relative cost-effectiveness advantage of 1-dose.

### 2.5 Epidemiological and natural history parameters

Age-standardised cervical cancer incidence and mortality rates were from GLOBOCAN 2022 version 1.1 (1): Kenya (incidence 32.8, mortality 21.4 per 100,000) and India (incidence 17.7, mortality 11.2 per 100,000). Stage distribution and five-year survival by stage were from Sankaranarayanan et al. (18). Natural history transition probabilities were derived primarily from high-income country studies given limited LMIC-specific data (19–23); all were varied in sensitivity analyses. Background mortality used WHO GHE 2021 life tables converted from 5-year to single-year probabilities using a constant hazard assumption. Parameter ranges reported in Table 1 were derived as follows: for epidemiological rates with published 95% confidence intervals (GLOBOCAN incidence, vaccine efficacy), ranges correspond to the reported CI bounds; for natural history transition probabilities without published uncertainty estimates (22, 23), ranges span ±50% of the central estimate, consistent with the ranges used in Prem et al. (6) and Jit et al. (14) for the same parameters; for stage distribution (Dirichlet), ranges correspond to the 25th–75th percentile of the pooled LMIC Sankaranarayanan et al. (18) data. All ranges were validated against published LMIC-specific distributions where available.

### 2.6 Cost parameters

All costs were expressed in 2024 USD. Vaccine unit price was USD 4.50 per dose (24); delivery cost was USD 2.00 per dose, consistent with the range reported for introduction-phase and routine LMIC HPV vaccination programmes [financial cost per dose: USD 0.60–3.41 at introduction, USD 0.31–3.77 at routine implementation; (25)], giving USD 6.50 per-dose programme cost. Total vaccination cost was USD 6.50 per 1-dose recipient and USD 13.00 per 2-dose recipient. CIN2/3 treatment cost was set at USD 75 per episode, representing a conservative estimate for LEEP or cryotherapy in low-income LMIC settings, consistent with published data reporting LEEP costs as low as USD 75–163 per patient in sub-Saharan Africa (26). The estimate was treated as approximate given limited country-specific primary costing data and was varied across a plausible range in sensitivity analyses (Table 1). ICC treatment costs by stage were adapted from Insinga et al. (35) using a two-step adjustment: first, US costs were scaled to LMIC levels using the ratio of LMIC composite to US health expenditure per capita from World Bank International Comparison Programme (ICP) 2017 data (PPP scaling factor: approximately 0.08) (31); second, PPP-adjusted values were inflated to 2024 USD using the World Bank GDP deflator series. Resulting costs were: Stage I, USD 1,050; Stage II, USD 2,450; Stage III, USD 3,050; Stage IV (palliative), USD 1,600. These estimates carry substantial uncertainty given the absence of published LMIC-specific stage-stratified cervical cancer treatment cost data; all costs were varied across plausible ranges in sensitivity analyses (Table 1). Treatment costs were applied as annual costs per health state cycle, discounted at 3% per annum. Productivity losses were excluded (payer perspective). The PPP scaling factor of approximately 0.08 was derived as the ratio of the LMIC composite (Kenya + India average) to US gross national income per capita at purchasing power parity from the World Bank International Comparison Programme 2017 round (Kenya: USD 4,330 PPP; India: USD 6,760 PPP; United States: USD 60,110 PPP; composite 0.080–0.084 depending on weighting). This scaling approach assumes that relative price levels for cervical cancer treatment inputs scale proportionally to overall GNI/PPP, which is a pragmatic approximation in the absence of cervical cancer–specific input price data for both countries; the resulting costs were subsequently varied across a ± 50% range in sensitivity analyses to characterise this parametric uncertainty.

### 2.7 Health outcomes

The primary health outcome was DALYs averted, comprising years of life lost (YLL) and years lived with disability (YLD). YLL per cycle = cancer deaths × remaining life expectancy × discount factor, using life expectancy at birth (68.0 years; WHO Life Tables 2023). YLD used GBD 2019 disability weights: CIN2/3 = 0.011; treated ICC (Stages I–III) = 0.288; terminal ICC (Stage IV) = 0.451 (27). Both components were discounted at 3% per annum.

### 2.8 Cost-effectiveness analysis

ICER = (Cost_strategy − Cost_reference) ÷ (DALYs_reference − DALYs_strategy). A negative ICER indicates a dominant strategy (simultaneously cost-saving and more effective). Because both strategies demonstrated dominance in the base-case, ICERs were supplemented by absolute cost savings and DALYs averted. WTP thresholds were USD 200/DALY (low-income) and USD 500/DALY (lower-middle-income) from Disease Control Priorities 3rd edition (28), preferred over GDP-based thresholds which overestimate real WTP in resource-constrained settings.

### 2.9 Sensitivity analyses

PSA used 10,000 Monte Carlo simulations (random seed: 20260101). Eleven parameters were sampled simultaneously: Beta distributions for probabilities (vaccine efficacy, natural history transitions, HPV 16/18 attribution, ICC disability weight) and Gamma distributions for cost parameters (delivery cost, ICC Stage III treatment cost). Distribution parameters were derived by method of moments from published point estimates and confidence intervals. Discount rate was fixed at 3% in PSA. OWSA varied eight parameters individually; results are presented as a tornado diagram. Pre-specified scenario analyses examined: second-dose dropout rates of 5, 15% (base-case), 30, 45% (each with 5,000 PSA simulations); 1-dose protection duration of 10 years, 20 years (base-case), and lifetime; discount rates of 0 and 5%; vaccine unit price ±50%. All analyses were conducted in R version 4.5.2 (dampack, tidyverse, ggplot2).

## 3 Results

### 3.1 Base-case cost-effectiveness

In the base-case analysis, both HPV vaccination strategies were dominant (simultaneously cost-saving and more effective) compared with no vaccination across a lifetime cohort of 100,000 girls (Table 2). Under no vaccination, the model projected 2,252 lifetime ICC cases, 2,165 ICC deaths, and 45,524 discounted DALYs. The total discounted cost of USD 18,090,009 represents cumulative lifetime treatment costs for CIN2/3 lesions and ICC across all four stages.

**Table 2:** Base-case cost-effectiveness results (Cohort *n* = 100,000 Girls aged 9 Years, LMIC composite).

Strategy	Total Cost (USD)	Total DALYs	ICC cases	ICC deaths	Cases prevented	Deaths prevented	DALYs averted	Incr. cost (USD)	ICER (USD/DALY)
No vaccination	18,090,009	45,524	2,252	2,165	Reference	Reference	Reference	Reference	Reference
1-Dose strategy	16,856,767	39,979	2,188	2,088	64	77	5,545	−1,233,242	Dominant^a^
2-Dose strategy	17,109,319	40,375	2,165	2,070	87	95	5,149	−980,690	Dominant^a^
2-Dose vs 1-Dose (incremental)	—	—	—	—	+23	+18	−396	+252,552	Extended dominance^b^

a Dominant = simultaneously cost-saving and more effective than no vaccination comparator; dominant in 99.3% (1-Dose) and 91.8% (2-Dose) of PSA simulations.

b Extended dominance: under the DALY-based cost-effectiveness framework, 1-Dose lies on the efficiency frontier because it both costs less and averts more DALYs than 2-Dose. The incremental ICER of 2-Dose vs 1-Dose is −USD 637/DALY; the negative sign indicates that 2-Dose is simultaneously more costly and less effective in DALY terms, not that it generates cost savings. This extended dominance ranking is conditional on the base-case 20-year single-dose protection duration assumption. Under a framework prioritising absolute cases or deaths prevented, 2-Dose prevents 23 additional cases and 18 additional deaths and may be preferred. LMIC composite = simple average of Kenya and India parameters. Time horizon: lifetime (age 9–99 years). Discount rate: 3% per annum for both costs and DALYs. Costs in 2024 USD. WTP thresholds: USD 200/DALY (low-income) and USD 500/DALY (lower-middle-income) from Disease Control Priorities 3rd edition (28).

The 1-dose strategy prevented 64 ICC cases and 77 deaths per 100,000 girls, averting 5,545 DALYs and generating a net cost saving of USD 1,233,242 relative to no vaccination (ICER: −USD 222/DALY). The 2-dose strategy prevented 87 ICC cases and 95 deaths, averting 5,149 DALYs and saving USD 980,690. The modest absolute reductions in cancer cases (2.8 and 3.9% for 1-dose and 2-dose, respectively) reflect the combined effect of programme-level effective coverage, vaccine efficacy against HPV 16/18, and the 72% HPV 16/18 attribution fraction; the maximum preventable fraction under base-case coverage assumptions is approximately 31% for 1-dose and 26% for 2-dose.

**Figure 2:**
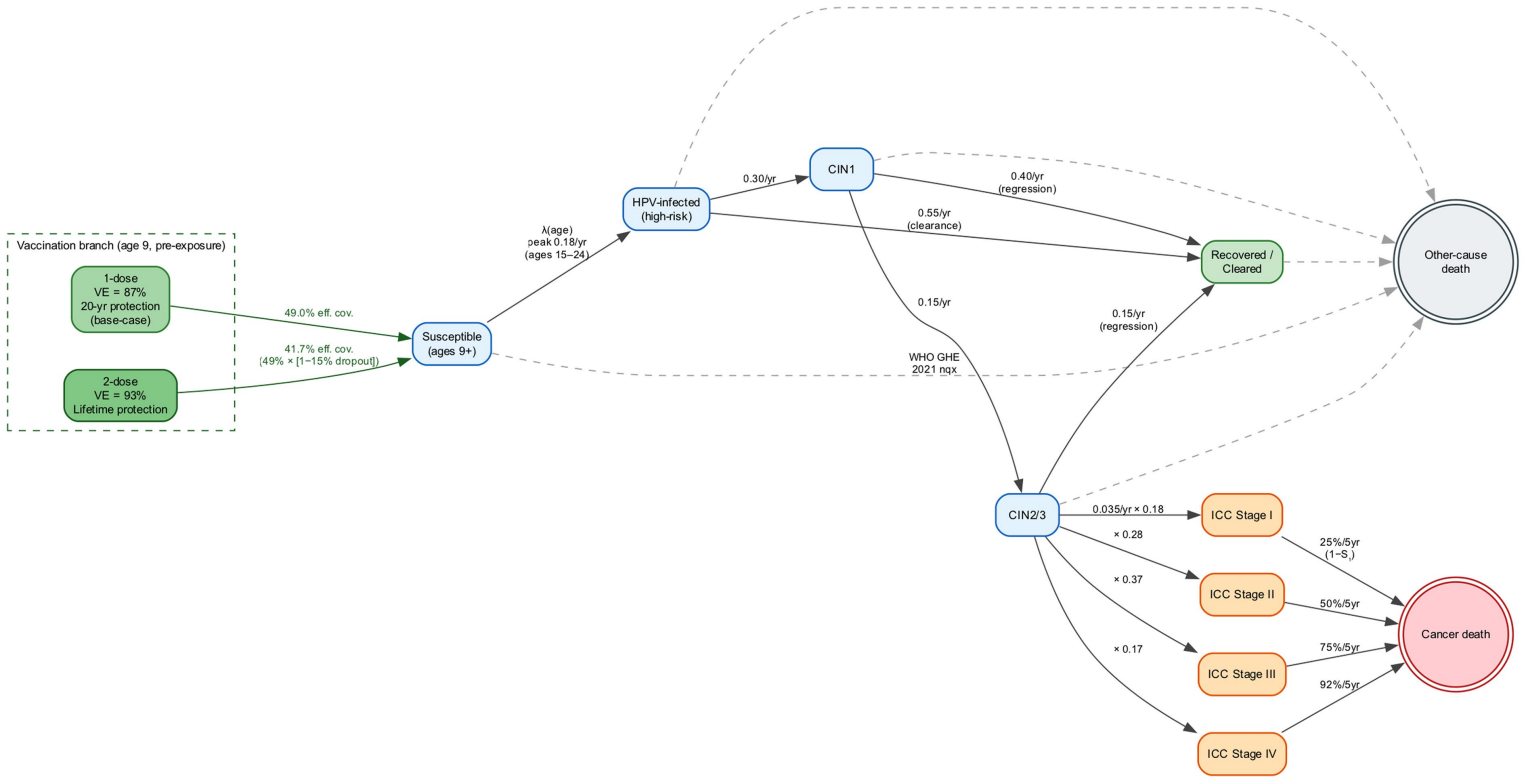
One-way sensitivity analysis tornado diagram for 1-dose HPV vaccination ICER versus no vaccination, Kenya–India LMIC composite. Each bar represents the ICER range when the indicated parameter is varied from its low to high extreme (all others at base-case). Dark blue = parameter at low value; light blue = parameter at high value. Dashed vertical line = base-case ICER (−USD 222/DALY). WTP thresholds: USD 200/DALY (solid red) and USD 500/DALY (dashed amber). Parameters sorted by influence (range) ascending. All ICERs remain negative (dominant) across all parameter ranges. 3% discount rate.

Despite preventing more absolute ICC cases and deaths, the 2-dose strategy averted 396 fewer DALYs than 1-dose (5,149 vs. 5,545). In the direct incremental comparison, 2-dose incurred an additional cost of USD 252,552 while averting fewer DALYs; under the DALY-based cost-effectiveness framework, 1-dose therefore lies on the efficiency frontier (extended dominance; see Table 2 footnote). The mechanism underlying this finding is explored further in Section 3.3. The divergence between DALY-based (1-dose dominant) and case-count-based (2-dose preventing more cases) strategy rankings is a structural consequence of the 20-year single-dose protection assumption in combination with the age-concentration of HPV acquisition. This mechanism, and its sensitivity to the protection duration assumption, is explored quantitatively in Section 3.3 and Figure 2 (tornado diagram).

### 3.2 Probabilistic sensitivity analysis

**Figure 3:**
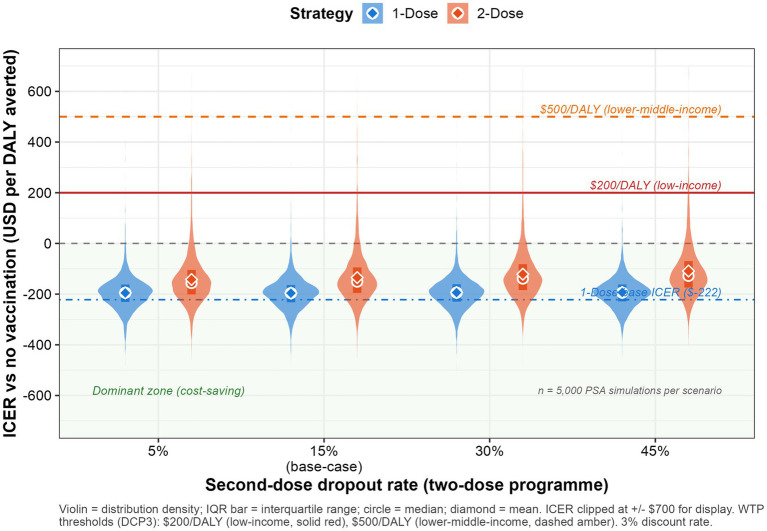
Incremental cost-effectiveness plane for 1-dose and 2-dose HPV vaccination versus no vaccination, Kenya–India LMIC composite. Each point represents one probabilistic sensitivity analysis (PSA) simulation (*n* = 3,000 randomly sampled from 10,000 total shown for clarity). Stars indicate deterministic base-case estimates. Points in the lower-right quadrant (positive DALYs averted, negative incremental cost) indicate dominant strategies. Willingness-to-pay thresholds: USD 200/DALY (low-income, solid red) and USD 500/DALY (lower-middle-income, dashed amber) from Disease Control Priorities, 3rd edition (28). Static direct-effect framework; 3% discount rate; costs in 2024 USD.

In the PSA (n = 10,000 simulations), the 1-dose strategy was dominant in 99.3% of simulations (mean ICER: −USD 195/DALY; 95% CrI: −USD 315 to −USD 64) and was cost-effective at both WTP thresholds in 100% of simulations (Supplementary Table S1; Figure 3). The 2-dose strategy was dominant in 91.8% of simulations (mean ICER: −USD 133/DALY; 95% CrI: −USD 284 to +USD 136). The upper 95% CrI boundary extending into positive territory (+USD 136) reflects residual uncertainty about 2-dose dominance under combinations of high delivery cost and low effective coverage. The 1-dose strategy had a more favourable ICER than 2-dose in 99.3% of PSA simulations.

Mean DALYs averted in PSA were 4,600 (95% CrI: 1,164–10,394) for 1-dose and 4,275 (95% CrI: 1,074–9,595) for 2-dose. These PSA means are lower than the deterministic base-case estimates because PSA samples the full joint distribution of parameter uncertainty, including scenarios with lower natural history progression rates. The incremental cost-effectiveness plane (Figure 3) shows PSA simulations for both strategies concentrated in the dominant quadrant, with the 1-dose cloud extending further along the DALYs-averted axis.

### 3.3 Age-distribution of protection and the DALYs–cases trade-off

The findings in this section are exploratory and derived using approximate state-transition calculations; they are presented as supplementary mechanistic explanation and should not be interpreted as primary model outputs. Readers primarily interested in the main cost-effectiveness results may proceed to Section 3.4. The finding that 1-dose averts more DALYs despite preventing fewer absolute ICC cases than 2-dose reflects a difference in the age-distribution of cancer prevention between strategies, which is itself a structural consequence of the 20-year base-case protection duration assumption. The 1-dose strategy, with protection expiring at age 29, concentrates cancer prevention in women aged 15–29—the peak HPV acquisition window—who have the greatest remaining life expectancy. The 2-dose strategy, with lifetime protection, distributes prevented cases across a wider age range including women aged 30 and older, in whom each averted cancer death contributes fewer years of life lost (YLL). DALY decomposition of the primary model output indicates that 95.8% of total DALYs are attributable to YLL, confirming that the age at which cancer is prevented is the dominant driver of DALY outcomes.

An exploratory age-band decomposition, derived using approximate state-transition calculations applied to the Markov trace outputs (Supplementary Table S3), generates estimates broadly consistent with this interpretation, though these should be regarded as illustrative approximations and should not be interpreted as primary model outputs or independent quantitative validation of the mechanism. Within the 15–24 age band, the exploratory decomposition suggests that approximately 62.9% of 1-dose DALYs averted and 61.1% of 2-dose DALYs averted originate from this age group. The DALY yield per averted case within this peak age band is similar between strategies (approximately 21.9 and 22.1 DALYs per case for 1-dose and 2-dose, respectively). The overall difference in DALY yield per case (86.4 vs. 59.2, derived from the primary Markov model output) is consistent with the expectation that 2-dose protection, extending beyond age 29, prevents cases at older ages where remaining life expectancy—and hence YLL per case—is shorter than for cases prevented during the 1-dose protection window.

An important model-structural feature is that the exploratory age-band decomposition (Supplementary Table S3) generates approximate estimates suggesting a small net excess of ICC cases in the 35–49 age band under 1-dose vaccination relative to no vaccination. This arises as an artefact of the hard protection expiry assumption at age 29: under the model structure, vaccinated individuals who are protected from cancer during ages 9–29 survive in greater numbers to ages 30 and beyond, where they remain susceptible to HPV infection after protection expires. This is a consequence of the specific assumption of abrupt rather than gradual protection expiry and does not represent a biological or clinical adverse effect of vaccination. The net result across all age bands in the primary Markov model output is 64 cases prevented and 5,545 DALYs averted for 1-dose (Table 2), which remains the authoritative estimate.

These results suggest a metric-dependent policy consideration that is structurally dependent on the assumed protection duration. Under a DALY-based framework and the 20-year base-case assumption, 1-dose was associated with greater DALY efficiency. Under a framework prioritising reduction of absolute cancer cases and deaths over the full lifetime, the 2-dose strategy offers advantages that are not captured in the DALY-based comparison. If single-dose protection is confirmed to extend beyond 20 years, the age-concentration mechanism would weaken and the two strategies would converge in both DALY and case-count outcomes.

### 3.4 One-way sensitivity analysis

The tornado diagram (Figure 2) shows the 1-dose ICER remained negative (dominant) across all parameter ranges explored. The two most influential parameters were 1-dose protection duration (ICER range: −USD 176 to −USD 243; influence range USD 67) and CIN1 → CIN2/3 annual progression rate (ICER range: −USD 175 to −USD 241; range USD 66). The CIN2/3 → ICC progression rate had minimal influence on the ICER (range USD 1.4), reflecting a structural feature of the incremental analysis: because vaccination prevents HPV infection rather than CIN2/3-to-ICC progression, changes in this parameter affect costs and DALYs proportionally across both arms, largely cancelling in the incremental calculation. Vaccine delivery cost was the third most influential parameter (range USD 26); 1-dose remained dominant even at the highest delivery cost explored (USD 4.00/dose).

### 3.5 Scenario analysis: impact of second-dose dropout rate

**Figure 4:**
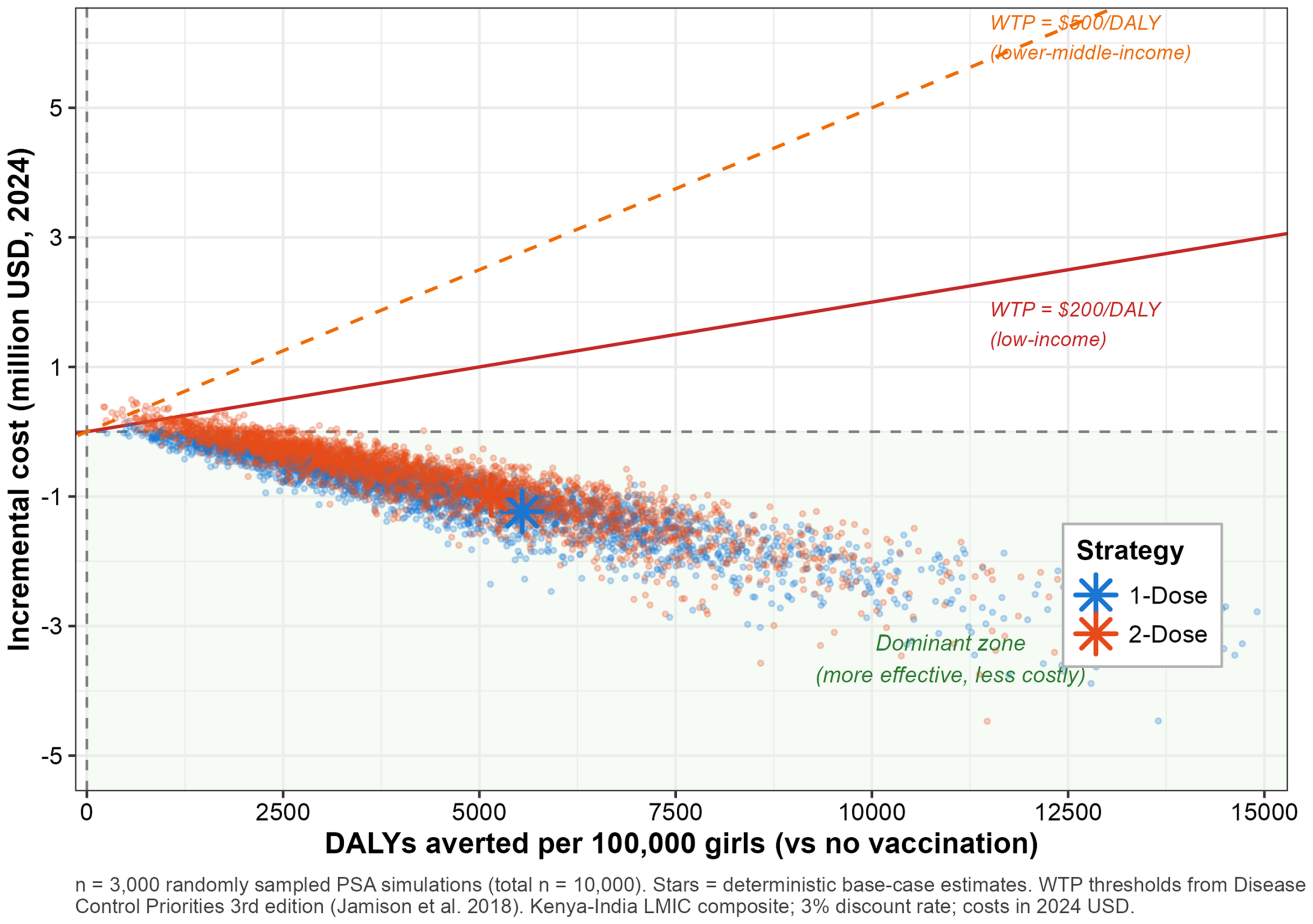
Distribution of incremental cost-effectiveness ratios (ICERs) for 1-dose and 2-dose HPV vaccination versus no vaccination, by second-dose dropout rate scenario (5, 15% [base-case], 30, 45%), Kenya–India LMIC composite. Each violin represents the PSA ICER distribution from 5,000 Monte Carlo simulations per scenario. Internal bar = interquartile range; circle = median; diamond = mean. ICER values clipped at ±USD 700 for display. Reference lines: USD 200/DALY (low-income WTP, solid red), USD 500/DALY (lower-middle-income WTP, dashed amber), 1-dose base-case ICER −USD 222 (blue dot-dash). 3% discount rate.

Across dropout scenarios from 5 to 45%, the 1-dose ICER was structurally invariant (mean range: −USD 193 to −USD 196; Figure 4, Supplementary Table S2). The 2-dose strategy showed systematic deterioration: mean ICER worsened from −USD 141/DALY at 5% dropout (effective coverage: 46.6%) to −USD 105/DALY at 45% dropout (effective coverage: 27.0% [= 0.49 × 0.55]). The upper 95% CrI increased from +USD 119 to +USD 189. The 1-dose strategy had a more favourable ICER than 2-dose in 99.0% of PSA simulations at 5% dropout, increasing to 100% at both 30 and 45% dropout. These results suggest that the effective coverage advantage of 1-dose, and the associated cost-effectiveness advantage, is largest in settings where second-dose completion is most uncertain.

## 4 Discussion

### 4.1 Principal findings

In this exploratory cost-effectiveness analysis of a Kenya–India LMIC composite, both single-dose (1-dose) and two-dose (2-dose) HPV vaccination strategies were associated with dominant cost-effectiveness compared with no vaccination across the majority of probabilistic simulations (99.3 and 91.8%, respectively), suggesting that HPV vaccination generates net cost savings regardless of schedule under the modelled coverage and cost assumptions. Under the base-case assumption of 20-year single-dose protection duration, the 1-dose strategy was associated with greater DALY efficiency per dollar invested, driven by a primary operational advantage of higher effective coverage through elimination of second-dose dropout (49.0% vs. 41.7%), and—under this specific protection duration assumption—a higher DALY yield per averted cancer case arising from the concentration of protection in young women aged 15–29 years (86.4 vs. 59.2 DALYs per case prevented).

It is important to emphasise that the DALY-based efficiency advantage for 1-dose over 2-dose is a property of the model structure under the 20-year protection assumption, not an empirical finding that would hold across all plausible assumptions. This finding depends on the hard expiry of 1-dose protection at age 29; if single-dose protection were 30 years or lifetime, the concentration-of-protection mechanism would weaken and the DALY efficiency advantage would diminish or reverse. Conversely, the 2-dose strategy prevents more absolute cancer cases and deaths across all scenarios examined, and this advantage would increase as dropout is reduced. These findings suggest that the DALY-based strategy ranking should be regarded as scenario-specific and assumption-dependent, and that any strategy preference communicated to stakeholders should explicitly state the protection duration assumption on which it is based. Readers interpreting these results for policy should explicitly note: the DALY-based preference for single-dose reported here is not a generalisable empirical claim, but a model-derived ranking that is conditional on the 20-year protection assumption; the two-dose strategy’s advantage in absolute case and death prevention is more robust across the scenarios examined and is the finding most stable to modelling assumptions.

A substantive finding is that the two strategies yield divergent strategy rankings depending on whether DALYs averted or absolute cases prevented is used as the primary outcome metric. This metric-dependent divergence has received limited attention in prior HPV vaccination cost-effectiveness analyses and represents a methodological contribution of this study.

It is equally important to emphasise the structurally robust finding in this analysis: across all protection duration scenarios examined (10-year, 20-year base-case, and lifetime), the two-dose strategy prevented more absolute invasive cervical cancer cases (by 18–31 cases per 100,000 girls) and more cancer deaths (by 13–22 deaths per 100,000 girls) than the single-dose strategy. This absolute-event advantage of the two-dose schedule is the finding least sensitive to modelling assumptions in the present analysis and should be weighted appropriately in policy discussions, particularly in settings where school-based delivery achieves low second-dose dropout.

### 4.2 Comparison with existing literature and incremental contribution

The cost-saving dominance of HPV vaccination versus no vaccination in LMIC settings is consistent with a growing body of published economic evidence. Multi-country analyses, including the 188-country modelling study by Prem et al. (6), have found that HPV vaccination strategies are cost-effective or cost-saving across the majority of LMICs under opportunity-cost-based thresholds. Our base-case ICER of −USD 222/DALY for 1-dose vaccination is within the range reported in comparable exploratory analyses. As with all static cohort models in this literature, our estimates capture only direct protection effects and should be interpreted as conservative lower bounds; dynamic transmission models incorporating herd immunity would be expected to yield more favourable cost-effectiveness estimates for both strategies.

The incremental contribution of the present analysis lies in three areas. First, we provide a DALY decomposition and an exploratory age-band analysis (Supplementary Table S3) generating approximate estimates broadly consistent with the interpretation that the age-distribution of cancer prevention is the dominant driver of DALY outcomes; these estimates are illustrative and should not be regarded as independent quantitative confirmation of the proposed mechanism. Second, we conduct systematic scenario analyses of second-dose dropout from 5 to 45%, showing that the effective coverage advantage of 1-dose scales monotonically with dropout rate. Third, we explicitly quantify and discuss the divergence between DALY-based and case-based strategy rankings, providing a framework for metric-transparent policy communication.

### 4.3 External validity of the LMIC composite setting

The Kenya–India composite was constructed as the simple average of country-specific epidemiological and cost parameters to give equal analytical weight to low-income and lower-middle-income contexts. This pragmatic design supports exploratory analysis of the general LMIC case, but it does not correspond to any real country’s parameter configuration. Several features limit direct applicability of our findings to individual settings. First, HPV genotype distribution differs between East Africa and South Asia [e.g., higher HPV 35 prevalence in sub-Saharan Africa; (19)], which may alter the effective 16/18 attribution fraction and vaccine-addressable burden. Second, the 49% coverage and 15% dropout assumptions represent averages that mask substantial within-country heterogeneity (e.g., urban vs. rural, school-based vs. facility-based delivery). Third, costs were adapted via PPP scaling rather than country-specific primary costing studies, and the true cost structure in either Kenya or India may differ materially from the composite. Consequently, the present analysis should be regarded as a framework for reasoning about the trade-offs between schedules, rather than as a country-level policy recommendation. Re-parameterisation with country-specific epidemiological, cost, and coverage data—and, where feasible, dynamic transmission modelling with country-calibrated sexual mixing structures—is required before the findings inform national programme decisions.

Our estimates are more conservative than some published analyses, reflecting a 1-dose VE of 87% (vs 97.5% infection-endpoint estimate) and a 20-year rather than lifetime base-case protection duration. Both are modelling choices made in the absence of long-term follow-up data. If longer protection duration is confirmed, the DALY efficiency advantage of 1-dose would diminish and both strategies would converge in effectiveness.

### 4.4 Second-dose dropout and programme design

The scenario analyses suggest that the effective coverage advantage of 1-dose vaccination—and therefore its cost-effectiveness advantage—scales with second-dose dropout rate. At 45% dropout, the effective coverage gap widens to 22 percentage points (49.0% vs. 27.0%), and the 2-dose upper 95% CrI extends to +USD 189/DALY, indicating that 2-dose dominance is not guaranteed across all parameter combinations at high dropout rates. Programmatic experience from Gavi-supported countries, as reviewed in WHO (5) and Ladner et al. (17), suggests that second-dose completion rates vary substantially and that real-world dropout may exceed the 15% base-case in contexts without school-based delivery. In settings with robust school-based delivery achieving near-complete two-dose coverage, the coverage gap narrows substantially and the 2-dose absolute case prevention advantage may become the decisive consideration.

### 4.5 Policy implications

Within the boundaries of this Kenya–India composite analysis and the static direct-effect framework employed, the findings are consistent with the rationale underlying the (5) recommendation that single-dose schedules represent an acceptable and potentially more coverage-efficient alternative to two-dose schedules in programmatic contexts where second-dose completion is uncertain. The cost-saving dominance of 1-dose versus no vaccination is robust across all parameter ranges and dropout scenarios examined within this framework, and its effective coverage advantage over 2-dose is largest in high-dropout settings where operational simplicity also favours single-dose delivery. Whether the DALY-based efficiency advantage holds in specific national contexts will depend on local dropout rates, emerging evidence on single-dose protection duration, and the health outcome metric prioritised. Final programme-level strategy rankings should be verified using country-calibrated dynamic transmission models that capture herd immunity effects.

Three practical considerations follow for programme planners. First, the cost-saving dominance of both schedules versus no vaccination suggests that HPV vaccination competes favourably for health system resources under opportunity-cost-based thresholds in both low-income and lower-middle-income country contexts. Second, programme planners achieving high 2-dose completion through school-based delivery may narrow the effective coverage gap; in such settings, the absolute cancer prevention advantage of 2-dose may outweigh the DALY efficiency advantage of 1-dose under current protection duration assumptions. Third, communicating programme impact using only absolute cases prevented may favour 2-dose, while reporting only DALYs averted may favour 1-dose under base-case protection duration assumptions. Transparent reporting of both metrics, with explicit acknowledgement of the assumptions driving their divergence, would help ensure that policy communications accurately reflect the structural conditions under which each strategy is preferred. The framing adopted in this paper—that both strategies should be compared using both DALY-based and case-based metrics, with the 20-year protection assumption explicitly acknowledged—is intended to support decision-makers in weighing operational feasibility (favoring 1-dose in high-dropout settings) against absolute event prevention (favoring 2-dose where coverage can be maintained). Neither schedule dominates the other across all assumption configurations, and the choice between them in any specific programmatic context should be informed by local evidence on second-dose completion, emerging long-term efficacy data on single-dose protection, and the health outcome metric prioritised by the national programme.

### 4.6 Limitations

Several limitations should be considered. First, and most importantly, the DALY-based efficiency advantage for 1-dose over 2-dose is structurally dependent on the 20-year base-case protection duration assumption, which is the single most influential parameter in the OWSA. This assumption is a modelling choice made in the absence of long-term efficacy data and could plausibly range from 10 years to lifetime. Results should not be interpreted as a robust, assumption-independent empirical finding in favour of 1-dose over 2-dose on DALY grounds.

Second, natural history transition probabilities were derived primarily from high-income country studies. Because such parameters affect both vaccinated and unvaccinated arms proportionally, the incremental ICER is likely stable in direction; nonetheless, this represents a structural limitation affecting the absolute magnitude of model outputs.

Third, the analysis was conducted for a composite setting derived as the simple average of Kenya and India parameters. This composite does not represent any specific country. Results should not be extrapolated to other LMIC settings without country-specific re-parameterisation and calibration.

Fourth, the static cohort model does not capture indirect protection through herd immunity; our results represent a conservative lower bound on population-level programme impact. Dynamic transmission models are needed to fully characterise programme-level effects. The magnitude of herd-immunity contribution depends on population coverage and sexual network structure. At the 49% coverage assumed in this analysis, published dynamic-model benchmarks suggest herd effects may increase DALYs averted by approximately 20–40% relative to the static estimate (12, 13). Because this uplift applies to both vaccination arms, the direction of the 1-dose vs. 2-dose ranking is unlikely to reverse under dynamic modelling at equivalent effective coverage, but the magnitude of the advantage may narrow as herd effects partially compensate for two-dose dropout-related coverage loss in the vaccinated subpopulation that receives only one dose (de facto 1-dose recipients within the 2-dose strategy).

Fifth, model face validity was assessed by comparing the no-vaccination arm projected lifetime ICC incidence against an annualised rate derived from the GLOBOCAN 2022 age-standardised incidence figure—a single-point consistency check rather than systematic internal or external validation. Age-specific predicted incidence was not compared against observed age-specific data, and no structural validation against independent datasets was performed. The model outputs should therefore be interpreted as order-of-magnitude indicative estimates, and conclusions should be regarded as conditional on the modelling assumptions rather than as confirmed empirical findings.

Sixth, ICC treatment costs were adapted from Insinga et al. (35) using World Bank PPP factors and GDP deflator adjustment—a pragmatic approximation; country-specific cost data would improve precision.

Finally, the analysis adopted a healthcare payer perspective, excluding productivity losses and out-of-pocket patient costs. A societal perspective would yield larger cost savings and more strongly dominant ICERs for both strategies.

## Data Availability

The model code and analytical scripts supporting the conclusions of this article are available from the corresponding author upon reasonable request.
