# Supplementary material for: Cost-effectiveness of single-dose versus two-dose HPV vaccination: a Markov cohort modelling analysis of a Kenya–India LMIC composite
Source: Front Public Health. 2026 Jun 4;14:1833764. doi: 10.3389/fpubh.2026.1833764 (PMC13277716; doi:10.3389/fpubh.2026.1833764)
Supplement: Supplementary file 1 [file Table_1.docx]

**Supplementary Tables**

**Table S1. Probabilistic Sensitivity Analysis Summary (n = 10,000 Monte Carlo Simulations)**

| **Comparison** | **Mean ICER (USD/DALY)** | **95% Credible Interval (USD/DALY)** | **% Simulations Dominant** | **% CE at USD 200/DALY** | **% CE at USD 500/DALY** | **n PSA Simulations** |
| --- | --- | --- | --- | --- | --- | --- |
| 1-Dose vs No Vaccination | −USD 195 | −USD 315 to −USD 64 | 99.3% | 100.0% | 100.0% | 10,000 |
| 2-Dose vs No Vaccination | −USD 133 | −USD 284 to +USD 136 | 91.8% | 99.4% | 99.9% | 10,000 |
| 2-Dose vs 1-Dose | −USD 3,913 | −USD 8,377 to +USD 4,849 | 89.8% | n/aᵃ | n/aᵃ | 10,000 |

*ICER = incremental cost-effectiveness ratio. 95% CrI = 95% credible interval (2.5th–97.5th percentile). Dominant = ICER < 0 (cost-saving and more effective). % CE = proportion of simulations cost-effective at stated WTP threshold. ᵃ % CE not applicable for 2-Dose vs 1-Dose comparison; the relevant metric is the proportion of simulations in which 1-Dose has a more favourable ICER than 2-Dose (99.3%). PSA parameters: vaccine efficacy (both schedules), all natural history transition probabilities, HPV 16/18 attribution, vaccine delivery cost, ICC Stage III treatment cost, DALY weight for treated ICC. Discount rate fixed at 3%, excluded from PSA. Random seed: 20260101. Kenya–India LMIC composite; static direct-effect framework; costs in 2024 USD.*

**Table S2. Scenario Analysis: Impact of Second-Dose Dropout Rate on Cost-Effectiveness (n = 5,000 PSA Simulations per Scenario)**

| **Second-Dose Dropout Rate** | **1-Dose Mean ICER (USD/DALY)** | **1-Dose 95% CrI (USD/DALY)** | **2-Dose Mean ICER (USD/DALY)** | **2-Dose 95% CrI (USD/DALY)** | **Interpretation** |
| --- | --- | --- | --- | --- | --- |
| 5% (low) | −USD 196 | −USD 317 to −USD 64 | −USD 141 | −USD 295 to +USD 119 | 1-Dose preferred in 99.0% of simulations. 2-Dose effective coverage: 46.6%. |
| 15% (base-case) | −USD 196 | −USD 317 to −USD 67 | −USD 133 | −USD 291 to +USD 134 | 1-Dose preferred in 99.7% of simulations. 2-Dose effective coverage: 41.7%. |
| 30% | −USD 194 | −USD 313 to −USD 63 | −USD 117 | −USD 275 to +USD 168 | 1-Dose preferred in 100% of simulations. 2-Dose effective coverage: 34.3%. |
| 45% (high) | −USD 193 | −USD 307 to −USD 69 | −USD 105 | −USD 263 to +USD 189 | 1-Dose preferred in 100% of simulations. 2-Dose effective coverage: 27.0%. Upper CrI substantially positive. |

*Each scenario holds second-dose dropout fixed at the stated value; all other PSA parameters sampled from assigned distributions. 1-Dose ICER is invariant to second-dose dropout because dropout affects only 2-dose effective coverage; 1-dose effective coverage remains 49.0% across all scenarios. 2-Dose effective coverage = 0.49 × (1 − dropout rate). CrI = 95% credible interval. Kenya–India LMIC composite; 3% discount rate; costs in 2024 USD.*

**Table S3. Exploratory Age-Band Decomposition of Prevented Cases, Deaths, and DALYs Averted by Strategy and Age Group versus No Vaccination: Approximate Estimates Derived from Markov Trace Outputs (Illustrative; Not Primary Model Outputs) (Cohort n = 100,000 Girls Aged 9 Years, Kenya–India LMIC Composite)**

**Important: Values in this table are derived from an approximate age-band calculation applied to the Markov cohort trace and should be treated as exploratory and illustrative only. They are not primary outputs of the Markov model; the authoritative base-case results are reported in Table 2 of the main text. Age-band values may not sum exactly to Table 2 totals owing to differences in calculation methodology.**

| **Age band (years)** | **1-Dose strategy (vs no vaccination)** | | | | **2-Dose strategy (vs no vaccination)** | | |  |
| --- | --- | --- | --- | --- | --- | --- | --- | --- |
|  | **Cases prevented** | **Deaths prevented** | **DALYs averted** | **DALYs per averted case** | **Cases prevented** | **Deaths prevented** | **DALYs averted** | **DALYs per averted case** |
| **9–14** | +44.5 | +2.0 | +119.7 | 2.7 | +40.4 | +1.8 | +108.7 | 2.7 |
| **15–24** | +159.4 | +69.9 | +3,488.9 | 21.9 | +142.5 | +63.0 | +3,145.4 | 22.1 |
| **25–34** | −1.8 | 45.8 | +1,934.7 | n/aᵃ | −0.6 | 40.1 | +1,696.1 | n/aᵃ |
| **35–49** | −46.6 | −17.9 | −467.8 | n/aᵃ | −27.0 | −8.7 | −217.5 | n/aᵃ |
| **50–64** | −8.4 | −9.5 | −145.4 | n/aᵃ | −5.8 | −5.7 | −85.0 | n/aᵃ |
| **65–99** | −1.8 | −1.8 | −6.0 | n/aᵃ | −1.5 | −1.3 | −4.1 | n/aᵃ |
| **All agesᵄ** | **+64ᵄ** | **+77** | **5,545** | **86.4ᵅ** | **+87ᵄ** | **+95** | **5,149** | **59.2ᵅ** |

*ᵃ n/a = DALYs per averted case not computed when net cases prevented ≤ 0 (see note below). Negative values in cases prevented, deaths prevented, and/or DALYs averted in older age bands are a model-structural artefact of the hard protection expiry assumption at age 29 for 1-dose vaccination: under the model structure, vaccinated individuals protected from cancer during ages 9–29 survive in greater numbers to older ages, where they remain susceptible to HPV infection after protection expires. This arises as a consequence of the specific assumption of abrupt rather than gradual protection expiry and does not represent a biological or clinical adverse effect of vaccination. The net result across all age bands in the primary Markov model (Table 2, main text) is 64 cases prevented and 5,545 DALYs averted for 1-dose, which remains the authoritative estimate. ᵄ Net totals from primary Markov model output (Table 2, main text); not the sum of age-band approximations. ᵅ DALYs per averted case for 'All ages' derived from primary model output (Section 3.3, main text). Base-case 1-dose protection duration: 20 years (expiry at age 29). 3% discount rate. Kenya–India LMIC composite; costs in 2024 USD.*
