# Supplementary material for: Cost-effectiveness of single-dose versus two-dose HPV vaccination: a Markov cohort modelling analysis of a Kenya–India LMIC composite
Source: Front Public Health. 2026 Jun 4;14:1833764. doi: 10.3389/fpubh.2026.1833764 (PMC13277716; doi:10.3389/fpubh.2026.1833764)
Supplement: Supplementary file 2 [file Table_2.docx]

**Supplementary Table S4. Benchmark comparison of model outputs with published HPV cost-effectiveness analyses applied to comparable LMIC settings**

| **#** | **Study** | **Setting** | **Model type** | **Primary reported outcome** | **Comparator** | **Direction consistent with present study?** |
| --- | --- | --- | --- | --- | --- | --- |
| 1 | **Present study** | Kenya–India LMIC composite | Static Markov cohort | Lifetime ICC per 100k = 2,252 (no vax); 2-dose averts 5,149 DALYs/100k; 1-dose ICER = −$222/DALY; 2-dose ICER = −$190/DALY (both deterministic base-case) ᶜ | vs no vaccination | — (reference) |
| 2 | **Prem et al., 2023 (BMC Med)** | 188 countries incl. Kenya, India | Hybrid: 3 dynamic + PRIME static | Across LMICs: 1-dose vaccination averts 32.5M (22.0–48.6M) cases over 100 years assuming 20-yr protection; HIC averts 4.8M (3.6–6.9M) | 1-dose vs no vaccination | ✓ Both schedules cost-effective in majority of LMICs under opportunity-cost thresholds |
| 3 | **Jit et al., 2014 (Lancet Glob Health)** | 179 countries incl. Kenya, India | PRIME static | HPV vaccination cost-effective in 156/179 (87%) countries; country-specific ICER reported for each | 2-dose vs no vaccination | ✓ Dominance/cost-effectiveness of 2-dose vaccination in Kenya and India settings |
| 4 | **Abbas et al., 2020 (Lancet Glob Health, PRIME updated)** | 179 countries updated demography/DALY weights/burden | PRIME static, updated | Updated country-specific ICERs; >85% of LMICs remain cost-effective under updated assumptions | 2-dose vs no vaccination | ✓ Updated estimates preserve dominance pattern in Kenya and India |
| 5 | **Drolet et al., 2021 (Lancet Infect Dis) ᵈ** | India, Vietnam, Uganda, Nigeria | HPV-ADVISE LMIC dynamic transmission | 2-dose routine (age 9–14) ICER range $28–$1,406/DALY across 4 LMICs; NNV range 78–381 | 2-dose vs no vaccination | ✓ 2-dose routine cost-effective in all 4 LMICs under WHO-CHOICE thresholds |
| 6 | **Umutesi et al., 2024 (Vaccines) ᵇ** | Kenya | Mathematical modelling + economic analysis | Baseline (no vax) incidence 26.0/100k/year; 1-dose 90% coverage ICER $197.44/DALY vs next non-dominated strategy; 2-dose 90% ICER $6,508.80/DALY vs next non-dominated | vs efficient frontier (not vs no vax) | ✓ 1-dose substantially more cost-effective than 2-dose at equivalent coverage in Kenya |

*ICC = invasive cervical cancer; DALY = disability-adjusted life year; ICER = incremental cost-effectiveness ratio; LMIC = low- and middle-income country; NNV = number needed to vaccinate; PRIME = Papillomavirus Rapid Interface for Modelling and Economics; WHO = World Health Organization.*

ᵃ Comparison metrics differ across studies because published HPV cost-effectiveness analyses report outcomes using different primary endpoints (per-cohort lifetime incidence, aggregate multi-year cases averted, country-specific ICERs against varying comparators). Direct per-100,000 lifetime ICC comparison was therefore not feasible across all cells. This table presents each study’s primary reported outcome with its original comparator, and summarises directional consistency with the present analysis under the shared qualitative finding that both single- and two-dose HPV vaccination schedules are cost-effective or cost-saving in LMIC settings under opportunity-cost-based willingness-to-pay thresholds.

ᵇ Umutesi et al. 2024 report ICERs against the efficient frontier (next non-dominated strategy), not against no vaccination; ICER values in this row are therefore not directly comparable to rows reporting ICER vs no vaccination. The directional consistency assessment is based on the rank ordering of 1-dose vs 2-dose strategies, which is comparable across both comparator frameworks.

ᶜ Deterministic base-case ICERs are shown. In the probabilistic sensitivity analysis (10,000 Monte Carlo iterations), mean ICER was −$195/DALY for 1-dose (dominant in 99.3% of simulations) and −$133/DALY for 2-dose (dominant in 91.8% of simulations); see Section 3.2 of main text.

ᵈ Drolet et al. 2021 received WHO funding and its modelling outputs directly informed the WHO Strategic Advisory Group of Experts on Immunization (SAGE) 2022 single-dose HPV vaccination recommendation (WHO Weekly Epidemiological Record 2022;97(50):645-672).
