# Supplementary material for: Cost-effectiveness of single-dose versus two-dose HPV vaccination: a Markov cohort modelling analysis of a Kenya–India LMIC composite
Source: Front Public Health. 2026 Jun 4;14:1833764. doi: 10.3389/fpubh.2026.1833764 (PMC13277716; doi:10.3389/fpubh.2026.1833764)
Supplement: Supplementary file 3 [file Table_3.docx]

CHEERS 2022 Checklist

|  | **Item** | **Guidance for Reporting** | **Reported in section** |
| --- | --- | --- | --- |
| **TITLE** | | |  |
| Title | 1 | Identify the study as an economic evaluation and specify the interventions being compared. | Title page: "Cost-Effectiveness of Single-Dose versus Two-Dose HPV Vaccination: A Markov Cohort Modelling Analysis of a Kenya–India LMIC Composite" |
| **ABSTRACT** | | |  |
| Abstract | 2 | Provide a structured summary that highlights context, key methods, results and alternative analyses. | Abstract section: structured Background, Methods, Results, and Conclusions subsections |
| **INTRODUCTION** | | |  |
| Background and objectives | 3 | Give the context for the study, the study question and its practical relevance for decision making in policy or practice. | Introduction §1–§4: cervical cancer burden in LMICs, single-dose evidence, economic evidence gaps, study objectives |
| **METHODS** | | |  |
| Health economic  analysis plan | 4 | Indicate whether a health economic analysis plan was developed and  where available. | ***Not Applicable.*** *This is an exploratory modelling study; no pre-registered health economic analysis plan was developed.* |
| Study population | 5 | Describe characteristics of the study population (such as age range, demographics, socioeconomic, or clinical characteristics). | Methods §2.2, paragraph 1: "hypothetical birth cohort of 100,000 girls aged 9 years" |
| Setting and location | 6 | Provide relevant contextual information that may influence findings. | Methods §2.1: Kenya–India LMIC composite setting; §2.5: country-specific epidemiological parameters |
| Comparators | 7 | Describe the interventions or strategies being compared and why chosen. | Methods §2.1: "three cervical cancer prevention strategies: no vaccination (reference), single-dose, and two-dose"; §2.4: Vaccination Strategies |
| Perspective | 8 | State the perspective(s) adopted by the study and why chosen. | Methods §2.1: "healthcare payer perspective" |
| Time horizon | 9 | State the time horizon for the study and why appropriate. | Methods §2.2: "lifetime (to age 99) using annual Markov cycles" |
| Discount rate | 10 | Report the discount rate(s) and reason chosen. | Methods §2.1: "discounted at 3% per annum"; §2.9: sensitivity at 0% and 5% |
| Selection of outcomes | 11 | Describe what outcomes were used as the measure(s) of benefit(s) and harm(s). | Methods §2.7 Health Outcomes: DALYs averted (primary), ICC cases prevented, deaths averted |
| Measurement of outcomes | 12 | Describe how outcomes used to capture benefit(s) and harm(s) were measured. | Methods §2.7: YLL (cancer deaths × residual LE) + YLD (prevalent cases × disability weight × duration) composition |
| Valuation of outcomes | 13 | Describe the population and methods used to measure and value outcomes. | Methods §2.7: GBD 2019 disability weights (Vos et al. 2020, ref 20); WHO GHE 2021 life tables (ref 23) |
| Measurement and valuation of resources  and costs | 14 | Describe how costs were valued. | Methods §2.6 Cost Parameters: vaccine unit price, delivery cost, CIN2/3 treatment cost, ICC treatment costs by stage |
| Currency, price date, and conversion | 15 | Report the dates of the estimated resource quantities and unit costs, plus the currency and year of conversion. | Methods §2.6: "All costs were expressed in 2024 USD"; PPP scaling from Insinga et al. using World Bank ICP 2017 factors and GDP deflator adjustment |
| Rationale and  description of model | 16 | If modelling is used, describe in detail and why used. Report if the model  is publicly available and where it can be accessed. | Methods §2.2 Model Structure: static Markov cohort model, 11 health states, annual cycles; §2.3 Calibration and Validation; Figure 1 (Markov state-transition diagram) |
| Analytics and assumptions | 17 | Describe any methods for analysing or statistically transforming data, any extrapolation methods, and approaches for validating any model used. | Methods §2.2–§2.9: model structure, natural history parameters from published sources, face validity via GLOBOCAN 2022 single-point check and benchmark comparison (§2.3), PSA/OWSA methodology (§2.9) |
| Characterizing heterogeneity | 18 | Describe any methods used for estimating how the results of the study vary for sub-groups. | ***Partially applicable.*** *Results §3.3 presents an exploratory age-band decomposition (Table S3); this is exploratory and does not constitute formal subgroup or heterogeneity analysis. Kenya vs India composite parameters were not stratified.* |
| Characterizing  distributional effects | 19 | Describe how impacts are distributed across different individuals  or adjustments made to reflect priority populations. | ***Not Applicable.*** *The cost-effectiveness analysis did not incorporate equity or distributional weighting. The LMIC composite setting itself reflects a priority-population focus, but no formal distributional analysis was performed.* |
| Characterizing uncertainty | 20 | Describe methods to characterize any sources of uncertainty in the analysis. | Methods §2.9 Sensitivity Analyses: PSA with 10,000 Monte Carlo simulations, OWSA across 8 parameters, scenario analyses for discount rate (0%, 5%) and dropout rate (5%–45%) |
| Approach to engagement with patients and others affected by the study | 21 | Describe any approaches to engage patients or service recipients, the general public, communities, or stakeholders (e.g., clinicians or payers) in the design of the study. | ***Not Applicable.*** *This is a modelling study based on publicly available aggregated data; no patient or stakeholder engagement was conducted.* |
| **RESULTS** | | |  |
| Study parameters | 22 | Report all analytic inputs (e.g., values, ranges, references) including uncertainty or distributional assumptions. | Table 1 Model Input Parameters: all parameter values, PSA distributions (Beta/Gamma/Triangular), ranges, and literature sources |
| Summary of main results | 23 | Report the mean values for the main categories of costs and outcomes of interest and summarise them in the most appropriate overall measure. | Results §3.1 Base-Case Cost-Effectiveness; Table 2: costs, DALYs, ICERs for all three strategies |
| Effect of uncertainty | 24 | Describe how uncertainty about analytic judgments, inputs, or projections  affect findings. Report the effect of choice of discount rate and time horizon, if applicable. | Results §3.2 PSA (Figures 2–3); §3.4 OWSA tornado diagram (Figure 4); §3.5 Dropout scenario analysis; Supplementary Tables S1–S2 |
| Effect of engagement with patients and others affected by the study | 25 | Report on any difference patient/service recipient, general public, community, or stakeholder involvement made to the approach or findings of the study | ***Not Applicable.*** *No patient or stakeholder engagement was conducted (see Item 21).* |
| **DISCUSSION** | | |  |
| Study findings, limitations, generalizability, and current knowledge | 26 | Report key findings, limitations, ethical or equity considerations not captured, and how these could impact patients, policy, or practice. | Discussion §4.1 Principal Findings; §4.2 Comparison with Literature; §4.3 External Validity and Dropout and Programme Design; §4.4 Policy Implications; §4.5 Limitations (7 limitations enumerated) |
| **OTHER RELEVANT INFORMATION** | | | |
| Source of funding | 27 | Describe how the study was funded and any role of the funder in the identification, design, conduct, and reporting of the analysis | Funding section (before References): "Shanxi Provincial Higher Education Science and Technology Innovation Program (Grant No. 2025L212). The funder had no role..." |
| Conflicts of interest | 28 | Report authors conflicts of interest according to journal or  International Committee of Medical Journal Editors requirements. | Conflicts of Interest section (before References): "The authors declare that the research was conducted in the absence of any commercial or financial relationships..." |

Husereau D, Drummond M, Augustovski F, de Bekker-Grob E, Briggs AH, Carswell C, Caulley L, Chaiyakunapruk N, Greenberg D, Loder E, Mauskopf J, Mullins CD, Petrou S, Pwu RF, Staniszewska S; CHEERS 2022 ISPOR Good Research Practices Task Force. Consolidated Health Economic Evaluation Reporting Standards 2022 (CHEERS 2022) Statement: Updated Reporting Guidance for Health Economic Evaluations. BMJ. 2022;376:e067975.

The checklist is Open Access distributed in accordance with the terms of the Creative Commons Attribution (CC BY 4.0) license, which permits others to distribute, remix, adapt and build upon this work, for commercial use, provided the original work is properly cited. See: [http://creativecommons.org/licenses/by/4.0/.](http://creativecommons.org/licenses/by/4.0/)
